# Dual-Mode Solidly Mounted Resonator-Based Sensor for Temperature and Humidity Detection and Discrimination

**DOI:** 10.3390/s24092877

**Published:** 2024-04-30

**Authors:** José Manuel Carmona-Cejas, Teona Mirea, Ricardo Hervás-García, Jimena Olivares, Marta Clement

**Affiliations:** CEMDATIC-ETSI Telecomunicación, Universidad Politécnica de Madrid, 28040 Madrid, Spain

**Keywords:** AlN, sensors, bulk acoustic wave, temperature control, humidity

## Abstract

Sensors based on solidly mounted resonators (SMRs) exhibit a good set of properties, such as high sensitivity, fast response, low resolution limit and low production cost, which makes them an appealing technology for sensing applications. However, they can suffer from cross-sensitivity issues, as their response can be altered by undesirable ambient factors, such as temperature and humidity variations. In this work we propose a method to discriminate humidity variations from the general frequency response using an SMR specifically manufactured to operate in a dual-mode (displaying two close resonances). The two modes behave similarly towards humidity changes (−1.94 kHZ/(%RH)) for resonance one and −1.62 kHZ/(%RH) for resonance two), whereas their performance under temperature changes is significantly different, displaying 2.64 kHZ/°C for resonance one and 34.21 kHZ/°C for resonance two. This allows for the decoupling process to be carried out in a straightforward manner. Frequency response is tracked under different humidity conditions, in the −20 °C to room temperature region, proving that this behavior is reproducible in any given environment.

## 1. Introduction

Electroacoustic resonators are one of the most relevant and well-established devices within the telecommunication industry, as they have become key components in current communication networks [1]. In addition to RF applications, these devices have been found to be promising as high resolution sensors for a wide variety of magnitudes and targets [2], such as temperature [3], pressure [4], different gas species [5,6,7] or even biosensing [8,9,10]. However, this versatility can lead to the observation of undesired effects, such as cross-sensitivity, or the response changes due to external ambient factors as temperature and/or humidity. Their use as gravimetric sensors relies on the shift experienced by the resonant frequencies when the targeted species are linked to the properly functionalized surface of the resonator.

Quartz crystal microbalances (QCMs) were the first family of resonators used in sensing applications [11], being routinely used for thickness monitoring in thin film deposition processes [12], or as gravimetric sensors to detect gas or biomolecules [13,14,15]. QCMs have also been studied as humidity sensors. Wang et al. [16] reported a QCM sensor with a PAA/PVA composite membrane active layer able to detect at room temperature relative humidity (RH) changes ranging from 0 to 95%. To prevent Q-factor values from dramatically dropping at high humidity concentrations, Yao et al. [17] added nanodiamond/graphene oxide nanocomposites on top of QCMs intended to detect humidity at room temperature.

Surface acoustic wave (SAW) devices have also been envisioned as temperature and humidity sensors [18], frequently taking advantage of their temperature coefficient of frequency (TCF) that is responsible for resonant (or anti-resonant) frequency shifts upon temperature changes. As temperature sensors, Borrero et al. [19] reported a 128° YX-cut, LiNbO_3_-based SAW sensor operating in the range between 50 and 200 °C, with a sensitivity of 87.81 ppm/°C. The TCF of the sensor can be tuned by a careful selection of the materials involved in the device to enhance its sensitivity to temperature. Alternatively, when the temperature influence is an undesirable effect, an appropriate selection of the materials can also help in reducing the TCF to values close to zero, with the addition of a SiO_2_ layer of appropriate thickness being the most common procedure for temperature compensation [20]. Regarding the operation of humidity sensors, Xuan et al. developed a graphene oxide-coated SAW humidity sensor showing high sensitivity in the 0.5% RH to 85% RH range, reaching 265 kHz/5%RH at room temperature [21]. In addition to these applications, researchers have proven the ability of SAW devices to work as sensors for many other magnitudes, such as pressure or biochemical species [22,23,24].

Thin film technology is also paramount for manufacturing acoustic resonator-based sensors. Film bulk acoustic resonators (FBARs), either in their free-standing or solidly mounted resonator (SMR) configuration, can operate the same way as QCMs and SAW devices, offering the advantage of their smaller size and higher operating frequency and quality factors. In 2007, Chiu et al. reported an FBAR device that could work either as a temperature or pressure sensor [25]. In 2013, He et al. took advantage of a dual-mode configuration FBAR sensor to detect temperature and pressure changes simultaneously [26]. In more recent years, researchers have been trying to improve FBARs’ sensitivity towards temperature, reaching sensitivity values up to 546 kHz/°C [27,28]. As for humidity detection with FBAR-based sensors, Liu et al. reported an FBAR sensor using a polyimide active layer and reaching sensitivities of up to 67.3 kHz/%RH at room temperature [29]. Later, the same group used the shifts in resonant frequency and in S11 magnitude to detect humidity and temperature variations [30].

However, the sensitivity of acoustic resonators to a large variety of physical magnitudes can lead to undesired effects like cross-sensitivity. Regarding the influence of temperature, different alternatives have been proposed for controlling or even suppressing it, such as the use of reference sensors or the tuning of the TCF of the whole structure by adding extra layers to the structure—a phenomenon called compensation [31,32]. It also has been demonstrated that manipulating film stress of the piezoelectric layer can lead to a reduction in temperature cross-sensitivity for some types of resonators [33].

In this work we propose another way to deal with this problem based on the use of SMRs specifically configurated to operate in a dual-mode configuration [34]. So far, FBARs in dual-mode configurations have been mainly studied as transducers for parallel sensing [34] and for enhancing sensitivity towards a single magnitude [35]. Beyond the sensor field, dual-mode resonators have been explored as potential candidates for RF filter applications [36]. In our previous work, it was proven that temperature effects could be discriminated by simultaneously tracking the evolution of both the resonant and anti-resonant frequency of an AlN-based SMR operating in the shear mode [37]. In this work we aim at detecting and decoupling humidity changes from the evolution of the frequency response of a resonator specifically designed to display two resonances at frequencies $f_{1}\;$and $f_{2}$ subjected to temperature and humidity changes.

This behavior could be described with the following equations:

(1) $$\begin{array}{c} \Delta f_{1}=TCF_{1}\cdot \Delta T+HCF_{1}\cdot \Delta (\mathrm{RH})\\ \Delta f_{2}=TCF_{2}\cdot \Delta T+HCF_{2}\cdot \Delta (\mathrm{RH}) \end{array}$$

where $\Delta f_{1,2}$ are the observed frequency changes, ΔT and Δ(RH) are the variations in temperature and relative humidity (%) experienced by the devices and $\mathrm{TC}F_{1,2}$, $\mathrm{HC}F_{1,2}$ are the temperature and humidity coefficients of frequency, respectively. Therefore, a single resonator in dual-mode configuration should allow quantifying humidity and temperature variations through a single measurement and decoupling one from the other.

To address this, in this work we manufactured SRMs displaying two close resonances and analyzed their frequency response when subjected to temperature and humidity sweeps. Their response was tracked over time and the influence of these two magnitudes was isolated and decoupled from each other.

## 2. Materials and Methods

AlN-based SMRs were manufactured in collaboration with Sorex Sensors Ltd. (Cambridge, UK) [38]. The devices consisted of an electroacoustic resonator made of a piezoelectric AlN film sandwiched between two electrodes and grown on top of an acoustic reflector similar to the one used in [39]. This configuration is formed by a series of alternating high and low acoustic impedance layers, with thicknesses equal to a quarter of the wavelength of the intended resonance, which helps to provide good acoustic insulation. Additionally, a 50 nm-thick Au layer was deposited on top of the devices to prevent the electrode from oxidating at high humidity concentrations and to prepare the sensor surface for further functionalization.

**Figure 1:**
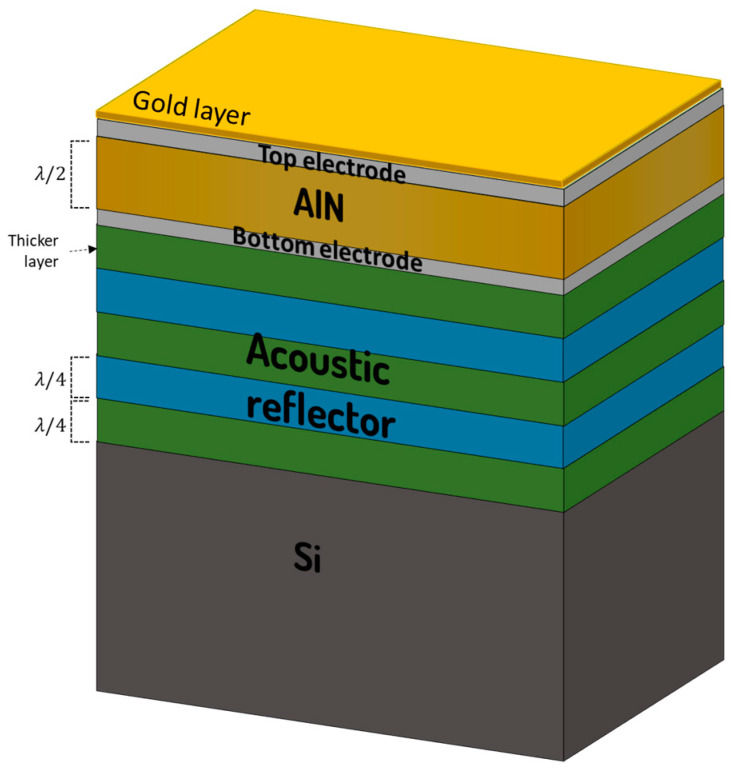
Diagram of the AlN-based SMR.

The fabricated SMRs displayed two longitudinal modes at 1.78 GHz and 2.33 GHz. This phenomenon was achieved by engineering the acoustic reflector, making the closest layer to the resonator thicker and thus generating an extra resonance at lower frequencies. These two resonances usually experience different behavior upon temperature variations [34]. Figure 1 shows the design diagram of the AlN-based SMRs employed for this work.

**Figure 2:**
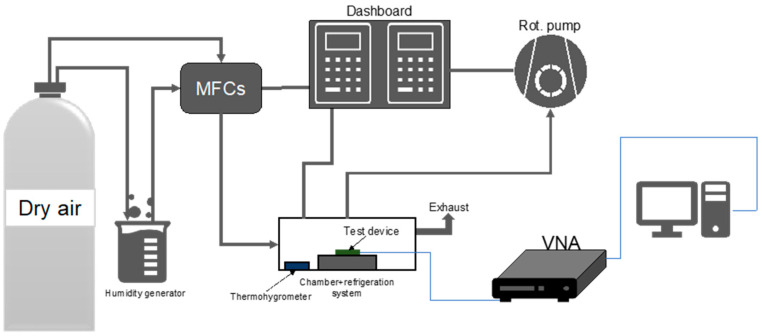
Experimental setup diagram.

The characterization of the frequency response of the devices subjected to temperature and humidity sweeps was carried out under the experimental setup shown in Figure 2. This consisted of a testing chamber connected to the vacuum system and to the gas inlet system. The chamber allowed for electrical characterization of the sensors through SMA connection to a Keysight (Santa Rosa, CA, USA) P9371A Streamline vector network analyzer (VNA) via an RF cable. To achieve and maintain temperatures below −20 °C in the chamber, a controllable Peltier module with its hot side sitting on top of a fluidic refrigeration system was utilized. The gas inlet system consisted of two gas lines; the first line was used for injecting dry air to the chamber, and the second line was used for humidity generation through a temperature-controlled bubbling system filled with deionized water. Total flow rates for the dry and humidified gases were controlled using mass flow controllers (MFCs). The different RH conditions were achieved by adjusting the ratios between dry and humidified gas, and measured using a hygrometer placed inside the chamber [40].

**Figure 3:**
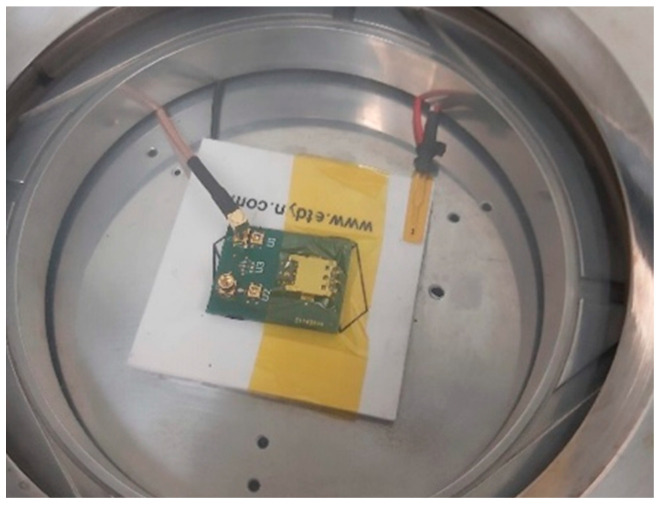
View of the inside of the characterization chamber. The SMR is bonded to the PCB and connected to a VNA via an RF cable.

After fabrication, the SMRs were bonded to a PCB (to ease the connection to the VNA from inside the testing chamber) and placed on the cold side of the Peltier module, as it can be observed in Figure 3. After evacuating the chamber with the rotary pump, the flows of dry and humid air were adjusted using the MFCs to set the desired RH conditions in the testing chamber. The exposure cycles consisted of controlled humid air exposure cycles followed by dry air exposure cycles. Each cycle was maintained for 15 min. The characterization was carried out by measuring the S parameters with the VNA and sending the data to a computer to process, using our own Labview 2020-designed software (National Instruments, Austin, TX, USA). The environmental conditions inside the chamber were measured using an NTC and humidity sensor. A set of measurements at different temperatures and RH conditions was carried out to prove the ability of the resonators to decouple the humidity and temperature contributions to the shift in resonant frequency.

**Figure 4:**
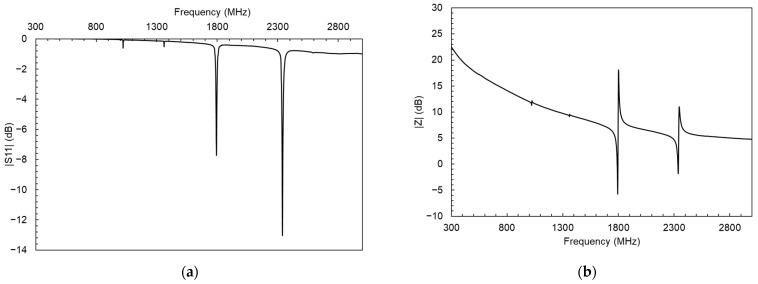
(**a**) SMR Frequency response measurement data collected from the VNA and (**b**) representation after transforming the S11 parameter data into electric impedance values.

The frequency response of the resonators was studied by measuring reflection parameter S11 with a VNA set in one-port configuration. A typical SMR frequency response can be observed in Figure 4a. It is possible to transform this type of measurement into electrical impedance values by using the following expression:

(2) $$Z=Z_{0}\frac{1+S_{11}}{1-S_{11}}$$

After this transformation, it is possible to identify the resonant and anti-resonant frequencies ($f_{r}$ and $f_{a}$, respectively) for each resonance. Frequency response after using Equation (2) is shown in Figure 4b. The positions of $f_{r}$ and $f_{a}$ for the two resonances are highlighted within the graph.

## 3. Results and Discussion

**Figure 5:**
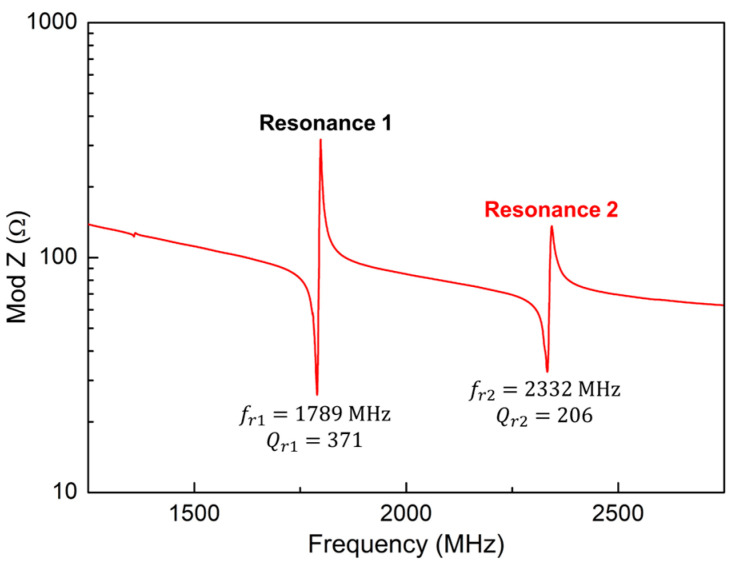
Typical frequency response of the SMRs employed as dual-mode sensors.

The typical frequency response of the SMRs used for this work is shown in Figure 5. Two resonances displaying (slightly) different properties appear separated by around 540 MHz. These peaks experience frequency shifts without interfering with each other over the wide range of temperatures and relative humidities envisaged in this work. The first mode has a resonant frequency of $f_{r}=1789\;\mathrm{MHz}$, an electromechanical coupling factor (${k_{\mathrm{eff}}}^{2}$) of about 1.15% and a resonant quality factor of $Q_{r}=371$, giving a figure of merit (FOM) of 426. For the second mode, $f_{r}=2332\;\mathrm{MHz}$, whereas ${k_{\mathrm{eff}}}^{2}=1.1\%$ and $Q_{r}=206$, resulting in FOM=227, indicating that both modes could be well-suited for sensing applications. A maximized FOM is not the aim of this work, as we have been focused on the temperature–humidity decoupling phenomenon.

The sensitivity of the two modes to temperature and relative humidity changes in the environment can be assessed through the corresponding variation coefficients in terms of frequency, as follows:

(3) $$\mathrm{TCF}=\frac{\Delta f}{\Delta T}$$

(4) $$\mathrm{HCF}=\frac{\Delta f}{\Delta \mathrm{RH}}$$

where Δf is the shift in resonant frequency, ΔT is the temperature variation, ΔRH is the relative humidity variation and TCF and HCF are the temperature and relative humidity frequency coefficients. These equations can also be normalized to the value of resonant frequency measured at initial time. Prior to operation in any given environment, these coefficients should be experimentally determined in order to have an SMR sensor with a proper calibration. Since our devices display two resonances, we have four coefficients in total to evaluate, regarding only resonant frequency.

TCF measurements were carried out by tracking the resonant frequency of the two modes while performing temperature sweeps at atmospheric pressure under constant humidity (RH=20%) conditions. The obtained coefficients were ${\mathrm{TCF}}_{1}=2.64\;\mathrm{kHz}/{}^{\circ}C$ for the first mode and ${\mathrm{TCF}}_{2}=34.21\;\mathrm{kHz}/{}^{\circ}C$ for the second mode. This supposes a variation of almost 32 kHz/°C between the two coefficients, with the second mode being much more sensitive to temperature changes than the first one, which displays a low variation coefficient.

**Figure 6:**
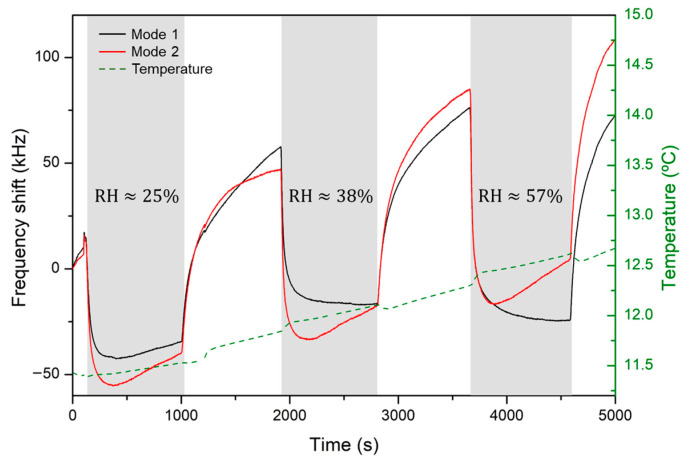
Frequency shift under different relative humidity atmospheres for resonance one (black) and resonance two (red). Temperature monitorization is displayed on the right axis.

Once the TCF was determined, the resonators were subjected to relative humidity changes in the 0 to ~65% range. During these experiments, the temperature was also tracked since slight temperature changes took place. The typical frequency shift of the two modes is shown in Figure 6. At first sight, both modes experience different shifts, although this can be explained by the small temperature changes undergone during data acquisition, as can be seen in the temperature variation measured and displayed on the right axis. Since the two modes behave differently under temperature changes, both frequencies experience different variations after each iteration. This also explains why, after each sensing iteration, both modes seem to move upwards in frequency.

**Figure 7:**
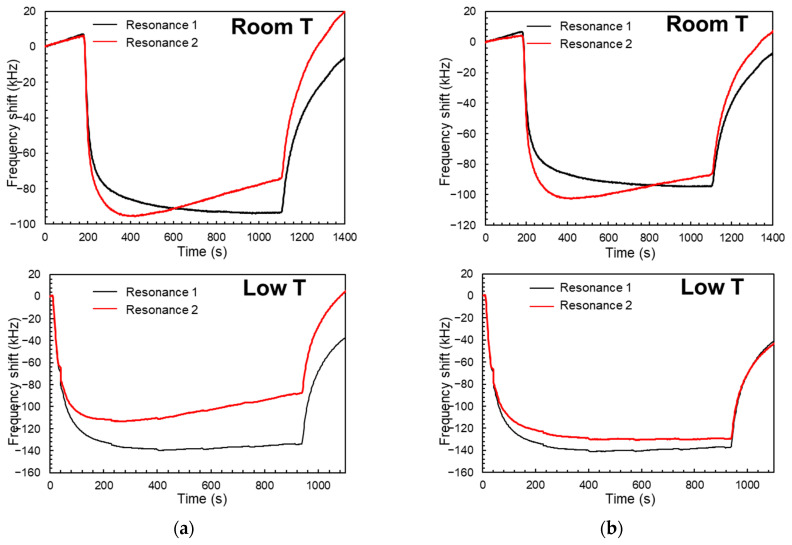
(**a**) Frequency shifts for a relative humidity variation of ~50% taken at 12 °C and at low temperature (~−2 °C), respectively, without keeping temperature constant; (**b**) same measurements after subtracting the temperature variation contribution to the frequency shifts.

Figure 7a shows the frequency response of the two modes for the same relative humidity variation taken at room temperature and at low temperature environments. The temperature changes during each measurement are also different, with the low temperature experiment being the one with higher temperature variation from start to finish, as it can be extracted from the higher difference between the two resonances. After applying a temperature-related correction, the two modes seem to behave more similarly under the two different temperature environments, as suggested by Figure 7b. This correction is no more than subtracting the frequency shift related to the temperature change during data acquisition and leaving just the humidity-related changes, as follows:

(5) $$\Delta f_{\mathrm{RH}}=\Delta f_{Total}-\mathrm{TCF}\text{∙}\Delta T$$

**Figure 8:**
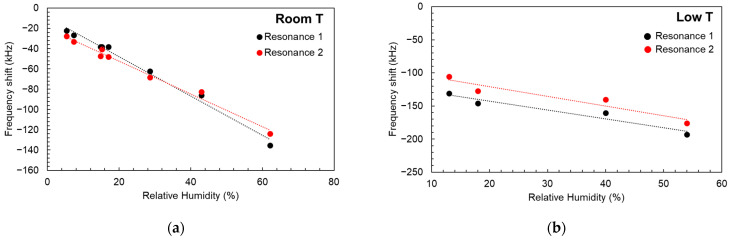
(**a**) HCF measurements for resonance one and resonance two within the range between 0 and ~65% RH, together with their linear regression fits performed at room temperature and (**b**) at below 0 °C temperature (−1.5 °C).

This approach works in the same manner for resonance one and resonance two using their calculated TCFs. With the resulting frequency shifts we can deduce the humidity coefficients for the two resonances. This calibration process is gathered in Figure 8a, where variations from 0 to ~65% are represented for the two resonances, together with their linear regression fits. Calculated HCF values were $\mathrm{HC}F_{1}=-1.94\pm 0.09\;\mathrm{kHz}/(\%\mathrm{RH})$ with a correlation coefficient of $R^{2}=0.99\;$for resonance one and $\mathrm{HC}F_{2}=-1.62\pm 0.08\;\mathrm{kHz}/(\%\mathrm{RH})$ and $R^{2}=0.98$ for resonance two. The variation between them, which in this case was 0.32 kHz/(%RH), can be neglected after considering the variation between temperature coefficients and thus implying a very similar sensitivity to humidity changes from both modes. A second set of calibration experiments was carried out at low temperatures (−1.5 °C) within the same RH range. The results are shown in Figure 8b. In this case, the calculated HCF values were $\mathrm{HC}F_{1}=-1.34\pm 0.26\;\mathrm{kHz}/(\%\mathrm{RH})$ with $R^{2}=0.93$ for resonance one and $\mathrm{HC}F_{2}=-1.48\pm 0.33\;\mathrm{kHz}/(\%\mathrm{RH})$ with $R^{2}=0.91$. The variation between coefficients was 0.13 kHz/(%RH). Therefore, the two resonances display the same relative behavior as that observed at room temperature.

We could try to decouple the influence of relative humidity from the response of the resonator as follows, combining the two expressions from Equation (1):

(6) $$\Delta f_{2}-\Delta f_{1}=\Delta T\left(TCF_{2}-TCF_{1}\right)+\Delta RH(HCF_{2}-HCF_{1})$$

**Figure 9:**
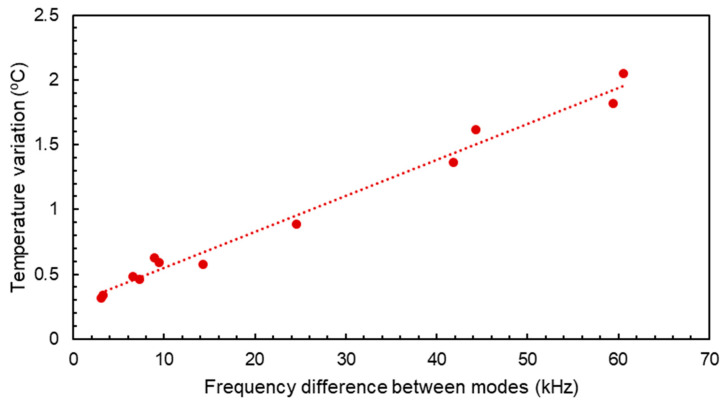
Difference in resonant frequency shift between resonance one and two for different temperature variations in the near-room-temperature range under arbitrary relative humidity concentrations.

**Figure 10:**
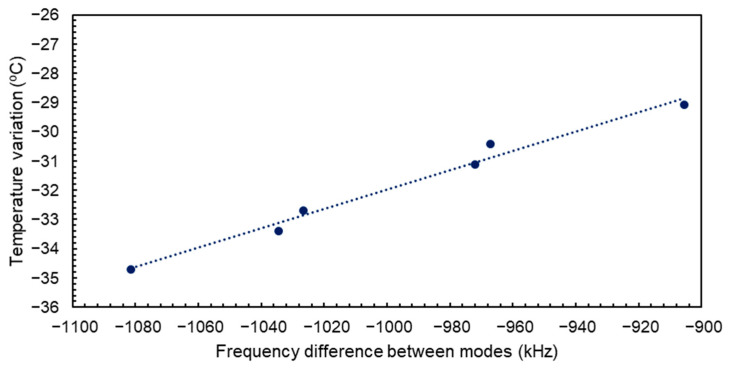
Difference in resonant frequency shift between resonance one and two for different temperature variations in the 17.5 to −18 °C range under arbitrary relative humidity concentrations.

And, after the results obtained from Figure 7 and Figure 8, we can assume that$\;\mathrm{HC}F_{1}=\mathrm{HC}F_{2}$. Thus, we can simplify this relation and obtain the following expression:

(7) $$\Delta T=\frac{\Delta f_{2}-\Delta f_{1}}{TCF_{2}-TCF_{1}}$$

which gives us a linear dependence between the temperature variations and the difference of frequency shifts between the two modes, independently from RH conditions. Figure 9 shows a series of measurements where temperature variations were recorded while the experimental conditions were set between 13 °C and 17.5 °C. The plotted frequency shifts represent the differences in resonant frequency between resonance one and resonance two. Additionally, arbitrary relative humidity concentrations between 0 and ~65% were injected into the testing chamber to force humidity-related frequency shifts for the two modes. Good linear dependence is observed between the frequency and temperature variations despite the variation in humidity atmospheres, leaving a linear regression parameter of a=0.02783±0.0011 °C/kHz, with a regression correlation coefficient of $R^{2}=0.99$. The calculated slope of this dependence from the TCF measured values would be ${\left(\mathrm{TC}F_{2}-\mathrm{TC}F_{1}\right)}^{-1}=0.0317\;{}^{\circ}C/\mathrm{kHz}$, which would lead to a difference of just 0.0039 °C/kHz, proving that there is a good agreement between the derived expression and the experimental results. The same experiment was carried out at a larger range of temperatures, going from 17.5 °C to −18 °C under arbitrary and different RH conditions. The results are gathered in Figure 10 together with their linear fit. In this case, the regression parameter is a=0.0331±0.0022 °C/kHz with a correlation coefficient of $R^{2}=0.99$. The difference between this parameter and the calculation from the TCFs is 0.0014 °C/kHz. The good correlation found in this case proves that this relation is independent from the temperature range and the humidity conditions in which the device is operating. If we combine the obtained results from Figure 9 and Figure 10, the resulting regression coefficient is a=0.03214±0.0001 °C/kHz with $R^{2}=0.99$. This coefficient only differs in 0.0004 °C/kHz from the calculated value, which is a variation of less than 2%.

Different humidity variations at different temperatures imply a different water concentration in atmosphere. Therefore, finding a similar behavior at room temperature and lower temperatures for the studied RH conditions suggest that the frequency variations measured for the two resonances are linked to RH variations, and not only to the absolute concentration of water in air. This behavior has also been reported for higher temperatures in [29], where the detection mechanism is not entirely gravimetric, but based on Young’s modulus variation of the sensing layer. For a gravimetric detection process with negative frequency shifts upon humidity exposure, a similar response for a higher temperature range is reported in [41].

## 4. Conclusions

In this work, the frequency response to both temperature and humidity changes was evaluated and calibrated for a dual-mode SMR sensor. Since the two modes react in the same consistent and predictable way to relative humidity changes, it is possible to decouple this effect from the sensor response, providing the ability to detect the contribution from other potential target analytes, as it was demonstrated with temperature changes.

The experimentation was conducted at room temperature and also at sub-zero temperatures, proving that the two modes are indeed sensitive to relative humidity changes and not purely to water concentration changes in air. Independently from the temperature conditions, the two resonances experienced the same variation of frequency after exposure to different RH conditions. This opens the possibility of just needing a single calibration procedure at any given temperature to work in any desired temperature and humidity condition.

As a potential application, the demonstrated reliability of the dual-mode SMRs in this work suggest that a single SMR could be enough to be used as a gravimetric sensor after decoupling both temperature and humidity effects from its frequency response. With this configuration, it could be possible to infer the frequency shift related to the detection of an extra target analyte by following a simple procedure, and without the necessity of a reference sensor.

## Data Availability

Data available upon request to the authors.
